# Compulsory Admission to Psychiatric Wards–Who Is Admitted, and Who Appeals Against Admission?

**DOI:** 10.3389/fpsyt.2019.00544

**Published:** 2019-08-09

**Authors:** Benjamin D. Arnold, Julian Moeller, Lisa Hochstrasser, Andres R. Schneeberger, Stefan Borgwardt, Undine E. Lang, Christian G. Huber

**Affiliations:** ^1^Klinik für Erwachsene, Universitäre Psychiatrische Kliniken Basel (UPK), Universität Basel, Basel, Switzerland; ^2^Department of Psychology, Division of Clinical Psychology and Epidemiology, University of Basel, Basel, Switzerland; ^3^Psychiatrische Dienste Graubünden (PDGR), Chur, Switzerland; ^4^Department of Psychiatry and Behavioral Sciences, Albert Einstein College of Medicine (AECOM), Bronx, NY, United States; ^5^Psychiatrische Universitätsklinik (PUK), Klinik für Psychiatrie, Psychotherapie und Psychosomatik, Universität Zürich, Zurich, Switzerland

**Keywords:** involuntary treatment, coercion, human rights, psychiatry, Switzerland

## Abstract

**Background:** When persons with a mental illness present a danger to themselves or others, involuntary hospital admission can be used to initiate an immediate inpatient treatment. Often, the patients have the right to appeal against compulsory admission. These processes are implemented in most mental health-care systems, but regulations and legal framework differ widely. In the Swiss canton of Basel-Stadt, a new regulation was implemented in January 2013. While the current literature holds some evidence for factors associated with involuntary admission, knowledge on who uses the right to appeal against admission is sparse.

**Aims:** The study aims to examine if specific sociodemographic and clinical characteristics are associated with involuntary admission and with an appeal against the compulsory admission order.

**Method:** Routine clinical data of all inpatient cases admitted during the period from January 2013 to December 2015 at the Psychiatric University Hospital Basel were extracted. Generalized estimating equation (GEE) analyses were used to examine the association of sociodemographic and clinical characteristics with “involuntary admission” and “appeal against compulsory admission order.”

**Results:** Of the 8,917 cases included in the present study, 942 (10.6%) were admitted involuntarily. Of these, 250 (26.5%) lodged an appeal against the compulsory admission order. Compared with cases admitted on a voluntary legal status, cases admitted involuntarily were older and were admitted more often during the nighttime or weekend. Moreover, involuntarily admitted cases had more often a principal diagnosis of a schizophrenia spectrum disorder. Patients from cases where an appeal was lodged were more often female, had more often Swiss nationality, and were more often diagnosed with schizophrenia spectrum disorder.

**Conclusion:** Despite legal changes, the frequency of involuntary admissions in the observed catchment area seems to be relatively stable across the last 20 years. The percentage of appeals has decreased from 2000 to 2015, and only comparably few patients make use of the possibility to appeal. Better knowledge of the regulations, higher social functioning, and lower insight into illness might be associated with a higher probability of lodging an appeal. Future research should examine if specific patient groups are in need of additional assistance to exert their rights to appeal.

## Introduction

In emergency situations with impending danger to patients or others, and in chronically ill patients—when leaving them untreated would pose a danger to themselves or others—psychiatry has to exert a difficult dual mandate, balancing mental health care for the individual patient with his right to autonomy and the protection of others (1, 2). In the context of involuntary admission and of involuntary treatment, this concept of beneficial coercion is currently critically discussed (3, 4).

These matters are complicated through the rising awareness that involuntary measures in psychiatry can have adverse effects (4, 5): For example, closed-door settings may increase the risk of escalation and aggression (4), involuntary measures may be (re)traumatizing for the patients (6), and decreased therapeutic atmosphere and patient–therapist relationship (7, 8) may have lasting detrimental effects on a patient’s motivation for treatment. Furthermore, even though involuntary measures like compulsory admissions may be well accepted by a majority of the public (9), they constitute a source for stigmatization of psychiatry and its patients with all accompanying detrimental effects (10). In addition, involuntary measures may not always provide the intended protection their use is based on: For example, it has been shown that treatment in a closed-door setting does not necessarily have the previously assumed positive effects on aggression, suicidality, and absconding (11–13).

Often, decisions become increasingly difficult because not only psychiatric but also legal and ethical aspects have to be considered. While structural prerequisites differ widely on international and even local levels (14–20), there is an ongoing discourse in current psychiatric research marking minimal standards for the decision to involuntarily admit mentally ill persons to inpatient treatment. In particular, the UN Convention on the Rights of Persons with Disabilities (CRPD) has promoted a critical discourse, stating that “the existence of a disability shall in no case justify a deprivation of liberty” (21). There also seems to be an agreement across the published literature that compulsory measures in psychiatry have to be considered only as measures of last resort when no other less restrictive alternatives are available (4, 22, 23). Accordingly, legal and structural regulations for compulsory measures have high requirements regarding assessment of the situation, documentation, quality control, and monitoring and, in general, include the right of the patients to appeal against decisions (3, 24, 25).

The Psychiatric University Hospital of Basel-Stadt follows a long-term strategy to promote open-door settings in psychiatry (23) and to decrease compulsory measures and stigmatization (10, 26, 27). Accompanying research could show that in the years 2011 onward, the frequency of seclusion and involuntary treatment could be decreased (22, 28–30) and that therapeutic atmosphere and patient satisfaction could be increased (7, 31) without noticeable detrimental effects on patients’ and public safety and the provision of mental health-care support (32). In addition, a new legal framework concerning involuntary admission was implemented beginning in January 2013. However, until now, the effects on involuntary admissions and on appeals against these admissions have not been a focus of research, and, in particular, literature concerning predictors of patients’ appeals against involuntary admission in general is sparse (33).

## Aim of Study

The current study aimed to examine the frequency of involuntary admissions and of appeals against them and if specific sociodemographic and clinical characteristics are associated with involuntary admission and appeals against the compulsory admission order. Based on the published literature, we hypothesized that known predictors of violence, self-harm, and poor insight are associated with involuntary admission and that admissions outside of regular working hours are associated with involuntary admission and the probability of appeals.

## Materials and Methods

### General Framework

The data examined in the current analyses were gathered during a longitudinal hospital-wide 3-year observational study. It was conducted at the Department of Adult Psychiatry, Psychiatric University Hospital of Basel-Stadt (UPK). This hospital provides psychiatric in- and outpatient health care for about 190,000 people in Basel and the surrounding areas. The hospital has an inpatient treatment capacity of 250–260 beds on 15 wards. All wards provide diagnosis-specific psychiatric and psychotherapeutic treatment. While there are other specialized psychiatric hospitals and institutions in the canton, the UPK is the only institution for the compulsory admission of inpatients. In addition, basic health care in Switzerland covers only treatment in the home canton. Thus, nearly all involuntarily admitted psychiatric patients from the hospital’s catchment area are admitted at the UPK.

### Legal Framework

The legal framework concerning compulsory admission and appeals against admission is formed by regulations from cantonal and federal civil law (Swiss Civil Code, ZGB) and has been revised multiple times in the past. In 1978, the Swiss federal law was adapted to comply with the European Convention on Human Rights (ECHR), and the basis for a *Confinement to Provide Medical Aid* (“Fürsorgerischer Freiheitsentzug,” FFE; Art. 397a–397f ZGB) was created and applied beginning in January 1981. The latest revision has been carried out in January 2013 with the establishment of a *Placement to Provide Medical Aid* (“Fürsorgerische Unterbringung,” FU; Art. 426–439 ZGB). The legal basis in cantonal civil law is provided by the *Law for the Protection of Children and Adults* (“Kindes- und Erwachsenenschutzgesetz”, KESG; last revised on 01.01.2013) (25).

Three main pathways can lead to compulsory admission (24): 1) When a person is deemed as being in need for mental health care despite objecting to treatment, a specially qualified public health officer (“Amtsarzt”) has to assess the case. If i) there is a mental illness or mental impairment, ii) an impending danger to the person himself or to others, or a severe case of—e.g., physical or social—neglect (34), iii) there is an indication for inpatient treatment in a psychiatric clinic, iv) there is no other less restrictive measure available and commensurability is preserved, the public health officer initiates involuntary hospitalization for up to 6 weeks (25). 2) Following the principles outlined previously, the *Department for the Protection of Children and Adults* (“Kindes- und Erwachsenenschutzbehörde”, KESB)—normally reacting to prior notice about dangerous situations—may initiate involuntary treatment with no limitations concerning the duration of treatment. 3) If a person who has voluntarily come into psychiatric treatment chooses to be dismissed, the treating psychiatrist may retain the person in treatment for up to 72 h if he presumes that the principles outlined previously are fulfilled. Within this time period, the patient either has to be dismissed or a public health officer has to assess if, indeed, all requirements for involuntary admission are fulfilled.

Independently from the pathway leading to compulsory admission, all patients have the right to appeal against the decision to commit them involuntarily. Hospital staff keeps the patients informed about their rights, assists patients with the procedure to appeal, or appeals on behalf of the patient if there are any signs that the patient objects to being hospitalized but is not able to appeal herself/himself. A specialized court (“Gericht für Fürsorgerische Unterbringungen”, FU-Gericht) including a presiding judge, an external psychiatrist, and an advocate for the patient disputes the case within a maximum time of 10 days after the appeal is issued and decides if the inpatient treatment can be terminated or if the decision for an involuntary hospitalization is upheld. The patient may appeal to the Swiss federal court (“Bundesgericht”) if there are objections to this decision.

### Study Population

For the current study, we included all inpatients admitted to the Department of Adult Psychiatry of the UPK between 01/2013 and 12/2015. Due to legal requirements, all admitted patients were aged 18 and older. No further inclusion or exclusion criteria were defined to ensure a naturalistic sample.

### Documentation and Management of Clinical Data

All data are recorded electronically by the responsible psychiatrists and psychologists using the provided clinical documentation system in its current version (Medfolio, Nexus AG, Villingen-Schwenningen, Germany). A broad data set has to be documented to ensure an optimal quality of clinical work and due to legal requirements of the *Swiss Federal Office for Statistics* (“Bundesamt für Statistik”, BfS) and the *Swiss National Association for Quality Development in Hospitals and Clinics* (ANQ). This includes data on age, gender, nationality, marital status, housing situation, occupational situation, and principal diagnosis according to International Classification of Diseases, 10th revision (ICD-10) (35) at discharge. Type of admission was categorized as “voluntary” and “involuntary,” and the decision to appeal against the compulsory admission order was recorded as “yes” or “no.” In addition, the time of hospital admission was extracted from the patient files. We classified cases as admitted “within regular working hours” if admitted from Monday to Friday between 8 am and 4:59 pm. Cases admitted at any other time were classified as admitted at “nighttime or [during the] weekend.”

All data were recorded during routine treatment and anonymized during extraction. Thus, according to current legal regulation, no approval from the local ethics committee was required for the current study. The current investigation complies with all national and international regulations, as well as with the Declaration of Helsinki in its current revision.

### Statistical Analyses

We investigated the association of sociodemographic and clinical characteristics with type of hospital admission applying a panel data analysis using generalized estimating equations (GEE) with the binary response variable “type of admission” (voluntary vs. involuntary) and age, sex, nationality, marital status, housing situation, occupational situation, time of hospital admission, and F0, F1, F2, and F3 diagnoses as predictors. Due to the dependency of our observations within subjects, we chose compound symmetry as our covariance structure in the model (36). We repeated this analysis for all involuntarily admitted cases with “appeal against compulsory admission order” (yes vs. no) as the binary response variable.

Multiple imputation was used to estimate and compensate missing values for GEE analyses (37). An alpha level of 0.05 determined statistical significance, and data analysis was carried out using IBM SPSS Statistics for Windows, Version 25.0 (released 2017; IBM Corp., Armonk, NY).

## Results

We included 8,917 cases in the present study (equaling a mean of 2,972 cases per year), who received inpatient treatment during the observation period in our clinic. Across all cases, 7,975 (89.4%) were admitted on a voluntary legal status, and 942 (10.6%) were involuntarily admitted. Of these, a total of 250 (26.5%) lodged an appeal against the compulsory admission order. Demographic and clinical descriptive information is presented in **Table 1**.

**Table 1:** Demographic and clinical descriptive information in voluntarily and involuntarily admitted patients and in patients using or renouncing their right to appeal the compulsory admission order.

Characteristic	Type of admission			Appeal against compulsory admission order		
	Voluntary	Involuntary	Total	Yes	No	Total
	*n* = 7,975	*n* = 942	*n* = 8,917	*n* = 250	*n* = 692	*n* = 942
Age (years)	45.61	48.36	45.9	47.77	48.57	48.36
**Gender**						
Male	3,829 (48.0%)	454 (48.2%)	4,283 (48.0%)	94 (37.6%)	360 (52.0%)	454 (48.2%)
Female	4,146 (52.0%)	488 (51.8%)	4,634 (52.0%)	156 (62.4%)	332 (48.0%)	488 (51.8%)
**Nationality**						
Other	2,443 (30.6%)	306 (32.5%)	2,749 (30.8%)	62 (24.8%)	244 (35.3%)	306 (32.5%)
Switzerland	5,532 (69.4%)	636 (67.5%)	6,168 (69.2%)	188 (75.2%)	448 (64.7%)	636 (67.5%)
**Marital status**						
Married	1,607 (20.2%)	102 (10.8%)	1,709 (19.2%)	19 (7.6%)	83 (12.0%)	102 (10.8%)
Separated/divorced	1,644 (20.6%)	153 (16.2%)	1,797 (20.2%)	45 (18.0%)	108 (15.6%)	153 (16.2%)
Widowed	341 (4.3%)	46 (4.9%)	387 (4.3%)	9 (3.6%)	37 (5.3%)	46 (4.9%)
Unmarried	3,960 (49.7%)	422 (44.8%)	4,382 (49.1%)	115 (46.0%)	307 (44.4%)	422 (44.8%)
Unknown	423 (5.3%)	219 (23.2%)	642 (7.2%)	62 (24.8%)	157 (22.7%)	219 (23.2%)
**Housing situation**						
Private residence	2,965 (37.2%)	306 (32.5%)	3,271 (36.7%)	102 (40.8%)	204 (29.5%)	306 (32.5%)
Living together with others	3,367 (42.2%)	214 (22.7%)	3,581 (40.2%)	59 (23.6%)	155 (22.4%)	214 (22.7%)
Assisted living	531 (6.7%)	80 (8.5%)	611 (6.9%)	10 (4.0%)	70 (10.1%)	80 (8.5%)
Hospitalized or in penal institution	323 (4.1%)	64 (6.8%)	387 (4.3%)	13 (5.2%)	51 (7.4%)	64 (6.8%)
Homeless	222 (2.8%)	45 (4.8%)	267 (3.0%)	13 (5.2%)	32 (4.6%)	45 (4.8%)
Other	73 (0.9%)	20 (2.1%)	93 (1.0%)	6 (2.4%)	14 (2.0%)	20 (2.1%)
Unknown	494 (6.2%)	213 (22.6%)	707 (7.9%)	47 (18.8%)	166 (24.0%)	213 (22.6%)
**Occupational situation**						
Employed	1,591 (19.9%)	73 (7.7%)	1,664 (18.7%)	18 (7.2%)	55 (7.9%)	73 (7.7%)
In education or civilian or military service	268 (3.4%)	11 (1.2%)	279 (3.1%)	6 (2.4%)	5 (0.7%)	11 (1.2%)
Other types of regular work	366 (4.6%)	32 (3.4%)	398 (4.5%)	10 (4.0%)	22 (3.2%)	32 (3.4%)
Retirement/disability pension	2,886 (36.2%)	378 (40.1%)	3,264 (36.6%)	97 (38.8%)	281 (40.6%)	378 (40.1%)
Unemployed	2,061 (25.8%)	170 (18.0%)	2,231 (25.0%)	45 (18.0%)	125 (18.1%)	170 (18.0%)
Unknown	803 (10.1%)	278 (29.5%)	1,081 (12.1%)	74 (29.6%)	204 (29.5%)	278 (29.5%)
**Time of hospital admission**						
Nighttime or weekend	2,619 (32.8%)	621 (65.9%)	3,240 (36.3%)	160 (64.0%)	461 (66.6%)	621 (65.9%)
Regular working hours	5,356 (67.2%)	321 (34.1%)	5,677 (63.7%)	90 (36.0%)	231 (33.4%)	321 (34.1%)
**Principal diagnosis (ICD-10)**						
F0 Organic, including symptomatic, mental disorders	343 (4.3%)	118 (12.5%)	461 (5.2%)	22 (8.8%)	96 (13.9%)	118 (12.5%)
F1 Mental and behavioral disorders due to psychoactive substance use	1,878 (23.5%)	177 (18.8%)	2,055 (23.0%)	36 (14.4%)	141 (20.4%)	177 (18.8%)
F2 Schizophrenia, schizotypal and delusional disorders	1,333 (16.7%)	361 (38.3%)	1,694 (19.0%)	127 (50.8%)	234 (33.8%)	361 (38.3%)
F3 Mood (affective) disorders	2,533 (31.8%)	151 (16.0%)	2,684 (30.1%)	41 (16.4%)	110 (15.9%)	151 (16.0%)
F4 Neurotic, stress-related and somatoform disorders	1,065 (13.4%)	55 (5.8%)	1,120 (12.6%)	7 (2.8%)	48 (6.9%)	55 (5.8%)
F6 Disorders of adult personality and behavior	609 (7.6%)	48 (5.1%)	657 (7.4%)	8 (3.2%)	40 (5.8%)	48 (5.1%)
Other psychiatric diagnosis	117 (1.5%)	13 (1.4%)	130 (1.5%)	3 (1.2%)	10 (1.4%)	13 (1.4%)
No psychiatric diagnosis	97 (1.2%)	19 (2.0%)	116 (1.3%)	6 (2.4%)	13 (1.9%)	19 (2.0%)

Values are given as number (percentage) for nominal variables and in mean ± standard deviation for continuous variables.

Of all included cases, the top four principal psychiatric diagnoses were mood disorder (30.1%), substance use disorder (23.0%), schizophrenia spectrum disorder (19.0%), and neurotic/stress-related/somatoform disorders (12.6%). However, in cases with involuntary admission, the top four primary ICD-10 diagnoses were schizophrenia spectrum disorder (38.3%), substance use disorder (18.8%), affective disorder (16.0%), and organic psychiatric disorder (12.5%). 1.3% of all cases and 2.0% of all involuntarily admitted cases did not receive a psychiatric principal diagnosis at discharge.

The results of the GEE analysis with “type of admission” as dependent variable are shown in **Table 2**.

**Table 2:** Generalized estimating equation (GEE) analysis with type of admission (admitted voluntarily or involuntarily) as dependent variable.

Characteristic	*B*	SE	df	*p*	95% CI
Age	0.012	0.0031	1	.000	0.005 to 0.018
Gender	0.021	0.0921	1	.817	−0.159 to 0.202
Nationality	−0.228	0.0968	1	.018	−0.418 to −0.038
Marital status	0.113	0.0434	1	.011	0.027 to 0.200
Housing situation	0.084	0.0439	1	.060	−0.004 to 0.172
Occupational situation	0.056	0.0364	1	.132	−0.017 to 0.129
Time of hospital admission	−1.166	0.0799	1	.000	−1.322 to −1.009
F0 principal diagnosis	1.180	0.1885	1	.000	0.810 to 1.549
F1 principal diagnosis	0.115	0.1454	1	.431	−0.170 to 0.400
F2 principal diagnosis	0.924	0.1328	1	.000	0.664 to 1.185
F3 principal diagnosis	−0.199	0.1344	1	.138	−0.463 to 0.064
Constant	−3.073	0.2949	1	.000	−3.652 to −2.494

CI, confidence interval; df, degrees of freedom; SE, standard error.

The GEE analysis suggested that “type of admission” (voluntary vs. involuntary) was significantly associated with age, nationality, marital status, time of hospital admission, and a principal diagnosis of an organic psychiatric disorder or a schizophrenia spectrum disorder. Cases admitted involuntarily were older, had less often Swiss nationality, were less often married, and were admitted more often during the nighttime or weekend hours, compared with cases admitted on a voluntary legal status. Moreover, involuntarily admitted cases had more often a principal diagnosis of an organic psychiatric disorder or of a schizophrenia spectrum disorder. However, we found no significant differences regarding gender, housing situation, occupational situation, a principal diagnosis of substance use disorder, and a principal diagnosis of an affective disorder in voluntarily and involuntarily admitted cases.

The results of the GEE analysis with “appeal against compulsory admission order” as dependent variable are shown in **Table 3**.

**Table 3:** GEE analysis with appeal against compulsory admission order (yes vs. no) as dependent variable.

Characteristic	*B*	SE	df	*p*	95% CI
Age	0.000	0.0055	1	.967	−0.011 to 0.011
Gender	0.587	0.1726	1	.001	0.249 to 0.925
Nationality	0.474	0.1949	1	.015	0.092 to 0.856
Marital status	0.032	0.0852	1	.711	−0.136 to 0.199
Housing situation	−0.066	0.0670	1	.327	−0.197 to 0.066
Occupational situation	−0.026	0.0740	1	.723	−0.173 to 0.120
Time of hospital admission	0.044	0.1720	1	.797	−0.293 to 0.381
F0 principal diagnosis	−0.174	0.3742	1	.642	−0.907 to 0.560
F1 principal diagnosis	0.043	0.3192	1	.892	−0.582 to 0.669
F2 principal diagnosis	0.884	0.2666	1	.001	0.362 to 1.407
F3 principal diagnosis	0.496	0.3117	1	.111	−0.114 to 1.107
Constant	−2.582	0.6416	1	.000	−3.842 to −1.323

CI, confidence interval; df, degrees of freedom; SE, standard error.

This second GEE analysis suggested that “appeal against compulsory admission order” (yes vs. no) was significantly associated with gender, nationality, and a principal diagnosis of schizophrenia spectrum disorder. Patients from cases where an appeal was lodged against compulsory admission were more often female, had more often Swiss nationality, and were more often diagnosed with schizophrenia spectrum disorder, when compared with cases where no appeal was filed. In detail, 127 (35.2%) of 361 involuntarily admitted cases with a schizophrenia spectrum disorder lodged an appeal against their compulsory admission order.

## Discussion

To our knowledge, this is the first study to examine appeals against compulsory admission within the current legal framework in Switzerland.

Compared with previous publications describing the same catchment area and hospital (33, 38), the number of cases per year increased from 2,319 in the year 2000 (33) to a mean of 2,972 in the years 2013–2015. As the number of beds and the size of the catchment area did not change relevantly, this can mainly be seen as a correlate of a decreasing mean duration of treatment. Lopez et al. (38) and Eichhorn et al. (33) further reported about 320 cases of involuntary admission per year equaling about 170 per 100,000 persons in the catchment area for the years 1993 and 2000. In the present study, there were a mean of 314 involuntary hospitalizations per year equaling 165 per 100,000 persons in the catchment area. Thus, the frequency of involuntary admission can be seen as nearly unchanged from 1993 to 2015, despite notable changes in legal regulation. Due to the rising number of cases treated in the UPK, the percentage of involuntary admission decreased from 13.8% in 2000 (33) to 10.6% in 2013–2015. This might have exerted a positive effect on the mean clinical severity of cases and on the therapeutic atmosphere, further supporting the positive effects of the introduction of an open-door strategy in the hospital (22, 28, 29). In total, the frequency of involuntary admissions in our study is within the range found across Europe, with a minimum of 12.4 per 100,000 inhabitants in Italy and up to 232.5 per 100,000 inhabitants in Finland and with considerable national and regional variations (20, 39).

While the frequency of involuntary hospital admissions remained relatively stable with reference to the population in the catchment area and showed a limited decrease in relation to the number of inpatient cases per year, the percentage of appeals showed a notable decline: whereas 50.0% of the affected patients appealed against the decision to admit them involuntarily in 2000 (33), only 26.5% appealed in 2013–2015. Changes in the legal situation are only one factor that could be associated with this decrease. In particular, the introduction of an open-door policy with improvements in ward atmosphere, patient–therapist–relationship, diagnosis specific treatment programs on the wards, and an intensive discourse with public stakeholders—especially the public health officers deciding on involuntary admissions—might be relevant factors explaining this decrease (23, 32).

Exploratory analyses of the clinical characteristics showed that 1.3% of all cases and 2.0% of all involuntarily admitted cases did not receive a psychiatric principal diagnosis at discharge. While there are legal regulations where patients involuntarily admitted to a psychiatric hospital have to be diagnosed with a psychiatric illness (40), there might be scenarios in the current legal regulation in Basel-Stadt where it may be allowed to commit persons without a primary psychiatric diagnosis, e.g., in cases with a “mental impairment” not fulfilling diagnostic criteria for an ICD-10 diagnosis from chapter F and with a severe case of physical or social neglect. Furthermore, these might be cases where—in the initial situation with a limited set of available information and within limited time in an emergency situation—a public health officer presumes that a psychiatric disorder can be diagnosed, but during the course of hospitalization and at discharge, this diagnosis can be ruled out.

When considering predictors of involuntary admission, there was a significant association with higher age, presumably corresponding with the increased percentage of persons with an organic psychiatric disorder in the involuntarily admitted patients. While, in other studies, male gender has been repeatedly found to be associated with involuntary admission due to its known connection with aggression and violence (41–43), this was not the case in the current study. This may be a correlate of the legal criteria for compulsory admission in Basel-Stadt that are focused not only on aggression but also on conditions like self-harm, suicidality, and neglect. The other factors associated with involuntary hospital admission are in line with the literature (2), as lack in social support (e.g., as statistically associated with marital status and foreign nationality), difficult access to regular mental health care (e.g., as statistically associated with foreign nationality), increased use of emergency mental health care outside of normal business hours, and lack of insight (e.g., in organic psychiatric disorders and schizophrenia spectrum disorder) are known to be associated with involuntary commitment (14, 20, 39, 44). In this context, it is unclear why a diagnosis of an organic psychiatric disorder was not statistically associated with an appeal against involuntary admission; this should be subject to future research.

Female gender, being of Swiss nationality, and having a principle diagnosis of schizophrenia spectrum disorder emerged as significant predictors of appealing against compulsory admission. This might be connected with an increased probability to appeal in cases with better knowledge of the legal regulations and system and in persons with better social functioning and skills (45). In addition, persons with low insight into their illness (e.g., in an acute phase of a schizophrenia spectrum disorder) might be more prone to appeal against an—in their view unjustified—admission or might show more opposition to inpatient treatment leading to an appeal on their behalf.

Contrary to our hypothesis that decisions made in an emergency setting might be connected with a higher probability to appeal, admission time had no significant association with the decision to appeal. From a clinical point of view, this is of note, as 66% of the cases with involuntary admission presented during nighttime or weekends. This suggests that the majority of involuntarily admitted patients are assigned to our clinic outside of normal working hours. In the present study, 160 (26%) of a total of 621 cases admitted outside regular working hours lodged an appeal, compared with 90 (28%) of the total of 321 cases admitted during regular working hours. The finding that admission time was no significant predictor of appeals may be interpreted as an indicator that the system in place in Basel-Stadt requiring professional external assessment by public health officers is able to provide highly qualified decisions on involuntary admission within and outside of regular working hours. In addition, it underlines the importance of an inpatient treatment setting that ensures that working toward a therapeutic alliance and shared decision making with involuntarily admitted patients is equally pursued during nighttime and on weekends as during normal working hours. If this would not be the case, it could be expected that patients admitted involuntarily outside regular working hours would lodge appeals against compulsory admission orders more frequently, indicating comparatively more disagreement with treatment. However, other interpretations of these findings cannot be ruled out. While they could indeed be the correlate of ensured treatment consent and satisfaction, there is, e.g., the possibility that patients expect a lower chance of success with regard to an appeal for admissions outside regular working hours and that this causes comparable rates of appeals with regular working hours despite lower agreement with the decision to initiate inpatient treatment.

## Strengths

The current study explores a novel and clinically important topic, enabling better understanding on who appeals against involuntary admission in psychiatry. Strengths of this study include a naturalistic design with broad inclusion and no-exclusion criteria, examining a hospital with nearly complete coverage of involuntary inpatient treatment for its catchment area, the relatively large sample size of 8,917 cases, and the applied statistical analyses. In addition, comparison data from the examined catchment area are available over a time period of more than 20 years, enabling examination of the longitudinal development of the frequency of involuntary admissions and of appeals.

## Limitations

As the GEE analysis method used in the current paper requires an adequate minimum sample size, some clinically interesting questions could not be examined, e.g., the predictors of a successful appeal. Furthermore, the current study used routine data, which enabled analysis of a relatively large dataset—on the other hand, some relevant information is not available from routine data and could therefore not be analyzed (e.g., length of involuntary commitment). In addition, the generalizability of the presented findings is limited due to differing legal regulations within Switzerland and in other nations.

## Conclusion

The frequency of involuntary admissions in the observed catchment area seems to be relatively stable, with about 170 cases per 100,000 inhabitants in 1993, 2000, and 2013–2015. The percentage of patients who use the possibility to appeal has decreased from 2000 to 2013–2015, and only comparably few patients lodge an appeal. Better knowledge of the regulations, higher social functioning, and lower insight into illness might be associated with a higher probability of appealing against involuntary admission. Future research should examine if specific patient groups are in need of additional assistance to exert their rights to appeal.

## Data Availability

The raw data supporting the conclusions of this manuscript will be made available by the authors, without undue reservation, to any qualified researcher.

## Ethics Statement

According to current legal regulation, no approval from the local ethics committee was required for the current study.

## Author Contributions

CH designed the study. BA and JM collected the data. BA, JM, LH, and CH analyzed and interpreted the data. BA, JM, and CH wrote the initial draft of the paper. AS, SB, and UL revised the manuscript for important intellectual content. JM and LH had full access to all the data in the study and take responsibility for the integrity of the data and the accuracy of the data analysis. All authors have contributed to, read, and approved the final version of the manuscript. BA and JM contributed equally.

## Funding

There was no funding provided for this research.

## Conflict of Interest Statement

The authors declare that the research was conducted in the absence of any commercial or financial relationships that could be construed as a potential conflict of interest.
